# The Assessment of Clinical Characteristics, Treatment Patterns, and Burden of Illness in Patients With Episodic and Chronic Migraine: A Cross-Sectional Study

**DOI:** 10.7759/cureus.59073

**Published:** 2024-04-26

**Authors:** Shahul Irfan, Fadhil Mohammed, Shahul Hameed, Vasupriya Ravi, Kashish V, Srimati S, Sashanga Arumugam, Bala Subramanian, Sreevinishaa Ravichandran, Umarani Ravichandran

**Affiliations:** 1 Internal Medicine, Government Medical College and Hospital Cuddalore, Chidambaram, IND; 2 Radiology, Stanley Medical College and Hospital, Chennai, IND; 3 Medicine and Surgery, Stanley Medical College and Hospital, Chennai, IND; 4 Medicine, Government Medical College and Hospital Cuddalore, Chidambaram, IND; 5 General Medicine, SRM Medical College Hospital and Research Centre, Chennai, IND

**Keywords:** chronic migraine (cm), episodic migraine, drug use pattern, cross sectional studies, migraine disability index scale (midas), migraine disorder

## Abstract

Introduction: Migraine is a prevalent and disabling primary headache disorder worldwide, causing significant years lost due to disability (YLD) and impacting various aspects of everyday life. Despite its high prevalence and substantial burden, there is a lack of comprehensive data on clinical patterns and management trends, in places like Tamil Nadu, India. This study aims and also fill gaps by investigating and analyzing the clinical characteristics, treatment patterns, and illness burden among patients with episodic migraine (EM) and chronic migraine (CM) in the state of Tamil Nadu.

Study: This cross-sectional retrospective study was conducted at the Department of Neurology, Madras Medical College, Chennai, over a three-month period starting from January 2024 to March 2024. The study included migraine patients aged 18 years and above who met the International Classification of Headache Disorders (ICHD)-3 criteria and took treatment at the department. Data were collected using patient interviews, medical records, and counseling sessions and using a pre-designed questionnaire. Patient demographics, clinical characteristics, symptom prevalence, prescription patterns, and illness burden were analyzed accordingly. The Migraine Disability Assessment (MIDAS) questionnaire was used to measure the burden of illness.

Results: The analysis involved 400 migraine patients, 92.5% of them having EM and 7.5% of them having CM. The mean age of patients was 37.5 years, with a predominance of females (73.5%). Patients with CM had having significantly higher average number of headache days per month when compared to those with EM. Tension-type headache (TTH) and medication-overuse headache (MOH) were more prevalent in those CM patients. Trigger factors include lack of sleep, bright light exposure, and stress. Comorbidities such as diabetes mellitus, obesity, and depression were significantly higher in CM patients. Acute treatment included NSAIDs and Triptans, while preventive therapy was more commonly used in CM patients. The mean MIDAS score was significantly higher in CM patients, which indicates greater disability.

Conclusion: The study provides valuable insights into the clinical characteristics, treatment patterns, and burden of illness among migraine patients in Tamil Nadu, India. Significant differences were observed between EM and CM patients, which highlights the need for comprehensive management strategies. Preventive therapy, lifestyle modifications, and comprehensive assessment of disability are all important in addressing the variable needs of migraine patients and also reducing the burden of illness. Further research is necessary to explore additional factors influencing migraine outcomes in this population.

## Introduction

Migraine is one of the common disabling primary headache disorders which is characterized by recurrent attacks of headache with a range of accompanying symptoms [1]. The Global Burden of Disease Study showed that migraine ranks as the second highest cause of years lost due to disability (YLD) worldwide, which has contributed to about 45.1 million total YLDs globally, next to low-back ache [2]. About 90% of individuals who experience headaches report a reduced ability to function normally, while 33% require bed rest during a headache attack [3]. It significantly disrupts educational activities, household work, occupational, family, and social responsibilities. Additionally, migraine is the second-highest contributor to neurological disease burden, following stroke [2]. Migraine is more prevalent in women when compared to men. A systematic review and meta-analysis which got published in 2017 provided an estimate of the average global prevalence of migraine at the community level, which was found to be 11.6% [4]. According to the Headache Classification Committee of the International Headache Society (IHS), individuals who experience ≥15 headaches per month for over three months, with at least eight of them meeting the criteria for a migraine attack or responding to acute migraine-specific medication, are classified as having chronic migraine (CM) and those with ≤14 headaches per month are classified as having episodic migraine (EM) (International Classification of Headache Disorders (ICHD)-3). Though the impact is severe with high prevalence and significant health-related issues associated with migraine, there is a lack of information regarding clinical patterns and management trends in India, especially in the state of Tamil Nadu. To the best of our knowledge, no studies have been conducted pertaining to the subject matter of the present study in Tamil Nadu. The goal of this study is to investigate and analyze the clinical characteristics, prescription patterns, and illness burden in patients with EM and CM. The objectives of the studies are as follows: (i) To investigate and analyze the clinical characteristics of migraine patients, including demographics, symptom prevalence, comorbidities, and trigger factors, in both EM and CM cases; (ii) To analyze treatment patterns among migraine patients, focusing on both acute and preventive therapies; (iii) To assess the illness burden associated with migraine, utilizing the Migraine Disability Assessment (MIDAS) questionnaire; (iv) To compare our results with other similar studies conducted in different population and locality.

## Materials and methods

Study design and population

This research is a cross-sectional retrospective study conducted at the Department of Neurology, Madras Medical College, Chennai for a period of three months from January 2024 to March 2024. The study was started after getting approval from the Institutional Ethics Committee, Madras Medical College. All migraine patients, regardless of gender and aged ≥18 years, who met the ICHD-3 criteria and took treatment or admission to the Department of Neurology were included in the study. Patients were briefed about the study details before enrollment. Relevant data were collected from patient interviews, medical records, and counseling sessions by filling out a pre-designed questionnaire. The data includes details about patient demographics, clinical characteristics, symptom prevalence, prescription patterns, and illness burden. The following patients were excluded from the study: (i) children and adolescents <18 years; (ii) pregnant and breastfeeding women; (iii) patients who refuse to participate or provide informed consent; (iv) individuals with a previous history of significant head trauma. The sample size was calculated with a confidence of 95% and a margin of error of 0.5. The sample size calculated was 139. The burden of illness was measured using the MIDAS questionnaire [5].

Data analysis

After de-identification, the collected data were quality-checked and finally entered into an electronic database. Patients were classified into two groups: EM (≤14 HDM) and CM (≥15 HDM). A descriptive statistical analysis was done to compare the patient demographics, clinical characteristics, treatment patterns, and disease burden between patients with EM and CM. Qualitative data were expressed as numbers and percentages, and quantitative data as means with standard deviation (SD). Two sample t-tests were used for the continuous variables and Chi-squared tests for categorical variables to compare between these two groups. A p-value of less than 0.05 was considered statistically significant. Statistical analysis was performed using the Statistical Package for the Social Sciences (IBM SPSS Statistics for Windows, IBM Corp., Version 29.0, Armonk, NY).

## Results

Our analysis involved a total of 400 migraine patients, of which 370 (92.5%) patients had EM and 30 (7.5%) patients had CM. The mean age of patients with migraine was 37.5 ± 14 years. The average number of migraine HDM was reported as 3.3 ± 2.5 with the EM group and 11.2 ± 7.7 with the CM group (p <0.05). The mean BMI in the EM group was 20.6 ± 3.4, whereas it was 25.1 ± 3.2 in the CM group (p <0.05). Among these participants, patients with EM and CM exhibited comparable characteristics (p >0.05). However, BMI and HDM were significantly higher among CM patients compared to those with EM (p <0.005) (Table 1).

**Table 1:** A comparison of patient characteristics between two groups BMI: body mass index; HDM: headache days per month ^a^ Chi-squared test ^b^ unpaired t-test

Variables	Episodic Migraine (n=370)	Chronic Migraine (n=30)	Total (n=400)	P-value
Age (years)	37.5 ± 14.2	38.5 ± 13.9	37.5 ± 14	0.852^b^
Females	270 (72.9%)	24 (80%)	294 (73.5%)	0.401^a^
Smoking	48 (10.8%)	4 (13.3%)	52 (13%)	0.954^a^
BMI (Kg/m^2^)	20.6 (3.4%)	25.1 (3.2%)	21.9 (3.3%)	0.0001^a^
HDM	3.3 ± 2.5	11.2 ± 7.7	4.1 ± 3.7	0.0001^b^

The migraine-related symptoms that patients considered troublesome in the EM group were unilateral headache (47.8%), bilateral headache (18.6%), throbbing/pulsatile (15.4%), preceded by an aura (19.7%), nausea (6.2%), vomiting (5.4%), aggravated by physical activity (7.5%), associated photophobia (89%), associated phonophobia (85.4%); and in the CM group: unilateral headache (36.7%), bilateral headache (30%), throbbing/pulsatile (50%), preceded by aura (23.3%), nausea (16.7%), vomiting (23.3%), associated photo-phobia (86.6%), associated phono-phobia (90%). All the clinical characteristics were comparable between EM and CM group (p >0.05) except, pulsating/throbbing headache, nausea, and vomiting which were significantly (p <0.05) more common in CM patients than EM patients (Table 2).

**Table 2:** A comparison of clinical characteristics among two groups ^a^ Chi-squared test

Variables	Episodic Migraine (n=370)	Chronic Migraine (n=30)	Total (n=400)	P-value
Preceding Aura	73 (19.7%)	7 (23.3%)	80 (20%)	0.635^a^
Unilateral Headache	177 (47.8%)	11 (36.7%)	188 (47%)	0.238^a^
Bilateral Headache	69 (18.6%)	9 (30%)	83 (20.8%)	0.131^a^
Throbbing/Pulsatile Headache	57 (15.4%)	15 (50%)	72 (18%)	0.00001^a^
Nausea	23 (6.2%)	5 (16.7%)	28 (7%)	0.03^a^
Vomiting	20 (5.4%)	7 (23.3%)	27 (6.8%)	0.0001^a^
Photo-phobia	330 (89%)	26 (86.6%)	356 (89%)	0.671^a^
Phono-phobia	316 (85.4%)	27 (90%)	343 (85.8%)	0.488^a^

In this study, we found that all the individuals had more than one triggering factor. Most commonly identified triggering factors in both migraine groups (EM and CM) were lack of sleep (78.7% and 73.3% respectively), bright light exposure (76.2% and 86.7%), psychological stress (70% and 80%), skipping meals (46.2% and 46.6%), fasting (16.2% and 16.7%), too much sleep (18.1% and 13.3) and menstruation (11% and 13.3%). All the triggering factors were comparable (p >0.05), but none showed a significant association with either condition (Table 3).

**Table 3:** A comparison of triggering factors between two groups ^a^ Chi-squared test

Variables	Episodic Migraine (n=370)	Chronic Migraine (n=30)	Total (n=400)	P-value
Lack of Sleep	291 (78.7%)	22 (73.3%)	313 (78.2%)	0.497^a^
Too Much Sleep	67 (18.1%)	4 (13.3%)	71 (17.8%)	0.510^a^
Stress	259 (70%)	24 (80%)	282 (70.8%)	0.246^a^
Bright Light Exposure	282 (76.2%)	26 (86.7%)	308 (77%)	0.190^a^
Menstruation	41 (11%)	4 (13.3%)	45 (11.3%)	0.707^a^
Skipping Meals	171 (46.2%)	14 (46.6%)	185 (46.3%)	0.962^a^
Fasting	60 (16.2%)	5 (16.7%)	65 (16.3%)	0.948^a^

Approximately half of all patients with EM and CM had comorbidities. The most common comorbid conditions observed in both migraine groups included diabetes mellitus (11.9% and 26.7% respectively), hypertension (17% and 16.7%), dyslipidemia (6% and 6.7%), obesity (10.5% and 36.7%), sleep disorders (10.5% and 50%), gastrointestinal problems (15.4% and 20%), respiratory problem (asthma, chronic obstructive pulmonary disease (COPD): 9.5% and 6.7%) and depression (12.4% and 33.3%). Among these comorbid conditions, diabetes mellitus, obesity, depression, and sleep disorders had a significant association with CM (p <0.05) (Table 4).

**Table 4:** A comparison of comorbidities between two groups ^a^ Chi-squared test

Variables	Episodic Migraine (n=370)	Chronic Migraine (n=30)	Total (n=400)	P-value
Sleep Disorders	39 (10.5%)	15 (50%)	54 (13.5%)	0.00001^a^
Diabetes Mellitus	44 (11.9%)	8 (26.7%)	52 (13%)	0.020^a^
Hypertension	63 (17%)	5 (16.7%)	68 (17%)	0.959^a^
Gastro-intestinal Problems	57 (15.4%)	6 (20%)	63 (15.8%)	0.506^a^
Dyslipidemia	22 (6%)	2 (6.7%)	24 (6%)	0.872^a^
Depression	46 (12.4%)	10 (33.3%)	56 (14%)	0.001^a^
Obesity	39 (10.5%)	11 (36.7%)	50 (12.5%)	0.00003^a^
Respiratory Illness	35 (9.5%)	2 (6.7%)	37 (9.3%)	0.617^a^

Most patients with EM (92.4%) and CM (96.7%) were on acute treatment for their migraine, whereas 37.8% of EM and 76.7% of CM patients were on preventive therapy for migraine (p <0.05). Among the patients on combined therapy, 16.2% had EM, while 73.3% had CM (p <0.05%). The fact that a higher percentage of patients with CM were on preventive and combined therapy suggests a significant association of preventive and combined therapy with CM. Among the CM group, there were no patients who did not take any treatment (Table 5).

**Table 5:** A comparison of treatment patterns between two groups ^a^ Chi-squared test

Variables	Episodic Migraine (n=370)	Chronic Migraine (n=30)	Total (n=400)	P-value
Acute Treatment	342 (92.4%)	29 (96.7&)	373 (93%)	0.389^a^
Preventive Treatment	140 (37.8%)	23 (76.7%)	158 (39.5%)	0.00003^a^
Both	60 (16.2%)	22 (73.3%)	78 (19.5%)	0.00001^a^
No Treatment	5 (1.4%)	0	5 (1.2%)	

Our survey also revealed that a significant proportion of patients with migraine utilized non-steroidal anti-inflammatory drugs (NSAIDs) as part of their acute treatment regimen. Specifically, 80.5% of individuals experiencing EM and 80% of those with CM were consuming NSAIDs. In contrast, a smaller percentage of patients in the EM and CM group were using triptans (6.5% and 13.3% respectively), and other medications (paracetamol, flunarizine, 5.4% and 6.7%). Medication-overuse headaches (MOH) in EM and CM groups were 4.3% and 26.7% respectively, whereas tension-type headaches (TTH) in EM and CM groups were 21.9% and 50% respectively (p<0.05) (Table 6).

**Table 6:** A comparison of acute treatment drug patterns and complications between two groups NSAIDs: non-steroidal anti-inflammatory drugs; MOH: medication overuse headache; TTH: tension-type headache ^a^ Chi-squared test

Variables	Episodic Migraine (n=370)	Chronic Migraine (n=30)	Total (n=400)	P-value
NSAIDs	298 (80.5%)	24 (80%)	338 (84.5%)	0.942^a^
Triptans	24 (6.5%)	4 (13.3%)	28 (7%)	0.157^a^
Others	20 (5.4%)	2 (6.7%)	22 (5.5%)	0.770^a^
MOH	16 (4.3%)	8 (26.7%)	24 (6%)	0.00001^a^
TTH	81 (21.9%)	15 (50%)	96 (24%)	0.00001^a^

In the realm of preventive therapies for migraine, a notable proportion of individuals, comprising 13% of those with EM and 16.7% with CM, were found to utilize valproic acid. Other medications used by EM and CM patients were propranolol (6.4% and 10% respectively), amitriptyline (7.8% and 13.3%), and pregabalin (5.7% and 3.3%). While a single preventive drug usage between EM and CM groups was comparable (p >0.05), a combination (>1 drug) of preventive drugs was consumed by 3% in EM group and 30% in CM group (p <0.05), depicting a significant association between combination of preventive drug usage and CM groups, but the combination therapy did not include a common drug regime between both the groups (Table 7).

**Table 7:** A comparison of drugs between two groups ^a^ Chi-squared test

Variables	Episodic Migraine (n=370)	Chronic Migraine (n=30)	Total (n=400)	P-value
Valproic Acid	48 (13%)	5 (16.7%)	53 (13.3%)	0.566^a^
propranolol	24 (6.4%)	3 (10%)	27 (6.8%)	0.460^a^
Amitriptyline	29 (7.8%)	4 (13.3%)	33 (8.2%)	0.292^a^
Pregabalin	21 (5.7%)	1 (3.3%)	22 (5.5%)	0.588^a^
Combination (>1 drug)	11 (3%)	9 (30%)	20 (5%)	0.00001^a^

In assessing the severity of migraine using the MIDAS questionnaire, the mean MIDAS score among individuals with EM was 11.2 ± 26.3, while among those with CM, it was 21.7 ± 21 (p <0.05). When classifying individuals based on the severity of migraine using the MIDAS questionnaire, it was found that 53.5% of those with EM and 30% of those with CM reported no disability. Additionally, the severity of disability among EM and CM groups was mild disability (25.1% and 6.7% respectively), moderate disability (13.2% and 23.3%), and severe disability (8.1% and 40%). The analysis revealed significant associations between higher MIDAS scores and CM (p <0.05). Mild and moderate disability were significantly associated with EM (p <0.05), whereas severe disability showed a significant association with CM (p <0.05) (Table 8).

**Table 8:** A comparison of the burden of migraine between two groups using the MIDAS questionnaire MIDAS: Migraine Disability Assessment Score ^a^ Chi-squared test ^b^ unpaired t-test

Variables	Episodic Migraine (n=370)	Chronic Migraine (n=30)	Total (n=400)	P-value
Mean MIDAS Score	11.2 ± 26.3	21.7 ± 21	12.1 ± 22	0.033^b^
No Disability	198 (53.5%)	9 (30%)	207 (51.8%)	0.005^a^
Mild Disability	93 (25.1%)	2 (6.7%)	95 (23.8%)	0.022^a^
Moderate Disability	49 (13.2%)	7 (23.3%)	56 (14%)	0.125^a^
Severe Disability	30 (8.1%)	12 (40%)	42 (10.5%)	0.00001^a^

## Discussion

In this retrospective population-based study, the demographic details of migraine patients were analyzed. Migraine is found to be more prevalent between the ages of 35 and 45 years [6]. This is consistent with our study where the mean age of migraine patients was 37.9 ± 14.0. Migraine prevalence peaking between 35 and 45 years could be due to hormonal fluctuations, stress, and lifestyle changes, aligning during this period, and influencing susceptibility to migraine attacks [7]. The present study showed a higher prevalence of migraine among females (73.5%) which reaffirms many previous studies [2,8,9].

Smoking is one of the modifiable risk factors for migraine [10]. Smoking increases cardiovascular risk through inflammatory mechanisms, that may also be implicated in the onset of migraine [11]. Most of the smokers in our study were males (>95%) and 13% were current smokers, 10.5% had a past smoking history and 76.5% were non-smokers. Though smoking as a risk factor was comparable, we could not find a significant association between smoking with EM or CM. The mean headache frequency per month was 3.3 ± 2.5 in EM and 11.2 ± 7.7 in CM. These values go in line with studies conducted by Ueda et al. and Lombard et al. [9,12].

Comorbidity profiles differed between patients with EM and CM. Diabetes mellitus, obesity, depression, and sleep disorders (insomnia, rapid eye movement (REM) behavior disorder) were significantly higher (p <0.05) among patients with CM, while hypertension, dyslipidemia, and respiratory and gastrointestinal problems were comparable (p >0.05). This aligns with previous research where depression was found to be more prevalent among CM patients and identified as a known risk factor for CM [13]. The International Burden of Migraine Study (IBMS) also noted higher rates of psychiatric disorders in CM compared to EM [14]. Higher rates of cardiovascular risk factors like hypertension, dyslipidemia, and obesity have been observed in CM patients compared to those with EM. Obesity, specifically, is recognized as a significant modifiable risk factor for CM [15].

Identifying trigger factors or precipitants is often advised as a fundamental approach to managing migraine. These factors, which increase the likelihood of headaches in the short term, play a crucial role in the treatment of migraine [16]. In the present study, the commonly identified triggers, which were comparable include lack of sleep, bright light exposure, stress, and skipping meals, while less recognized triggers encompass excessive sleep, fasting, menstruation, caffeinated foods, and skipping meals.

In this study, approximately one-fourth of the patients had migraine aura (20%). The majority of them had visual symptoms (83%), followed by auditory, sensory, and language disturbances. Research conducted by Singla et al. similarly highlighted visual aura as the most prevalent symptom, followed by sensory aura, and occasionally, speech and language disturbances, with motor aura being less common [17]. Pain-related symptoms were the most troublesome for patients with either EM or CM. While the characteristics of migraine symptoms were similar between the two groups, throbbing/pulsatile headache was significantly associated with CM (p <0.05). Previous studies show that the prevalence of pulsatile headache in CM is about 80% [18]. Migraine entails various accompanying symptoms which include nausea, vomiting, neck stiffness, dizziness, irritability, and allodynia [19]. In our study, nausea and vomiting were the most common accompanying symptoms significantly associated with CM, while symptoms like photophobia and phonophobia were comparable between EM and CM.

Both oral NSAIDs and triptans are recommended for treating migraine attacks, as advised by the European Federation of Neurological Societies. In our study, prescriptions for acute treatment in patients with either EM or CM primarily included NSAIDs (84.5%) and triptans (7%), aligning well with European Headache Federation guidelines. The most commonly used NSAID was Aceclofenac (76%), followed by diclofenac (15%) and naproxen (5%). In the present study, there was a significant association with the usage of preventive drugs in CM patients. Nearly 77% of CM patients in our study were on preventive medications. Among preventive drugs, valproate (13%) was the most commonly used drug in both EM and CM groups followed by amitriptyline (8.2%), propranolol (6.8%), and pregabalin (5.5%). Studies have demonstrated that beta blockers are effective in reducing attack frequency by up to 50% [20]. Among antidepressants, amitriptyline is the most commonly utilized drug for migraine management. This is not in accordance with many studies [15] where topiramate is the most commonly used first-line preventative drug. A research trial conducted by Afshari et al. showed a better reduction of headache frequency with topiramate than with valproate [21]. The higher prevalence of valproate and amitriptyline usage among migraine patients in our area is attributed to the elevated cost of topiramate compared to valproate and amitriptyline. The other attributable reason may be the lack of supply of topiramate in government hospitals in our locality. Our survey also found that the prevalence of MOH and TTH was significantly higher in CM patients. MOH arises from the frequent treatment of headaches, leading to withdrawal headaches. Dysfunctions in receptor and enzyme physiology contribute to MOH, potentially causing tolerance and dependence. Tolerance may result in physical dependence, manifesting as withdrawal symptoms upon discontinuation of acute medications [22].

The MIDAS questionnaire assesses disability or limitations caused by headaches in three key domains: paid work, household work, and non-work or social activities. It quantifies not only missed days but also days with significantly reduced productivity in easily understandable units (lost days). It classifies patients into four groups based on the level of severity: no disability, mild disability, moderate disability, and severe disability [23]. In the scoring system, severe disability was significantly associated with CM, while no disability and mild disability were significantly associated with EM. This finding is consistent with studies conducted in Malaysia, Taiwan, and Italy [24-26].

Strengths

The research offers comprehensive insights into migraine by examining clinical characteristics, treatment patterns, and illness burden in 400 patients with EM and CM in Tamil Nadu, India. Utilizing established diagnostic criteria and robust data collection methods, the study provides reliable findings. Statistical analysis reveals significant differences between EM and CM, highlighting variations in symptom prevalence, comorbidities, and treatment approaches. The study’s clinical relevance underscores the need for tailored management strategies, emphasizing preventive therapies and lifestyle modifications to alleviate the burden of illness in migraine patients. Overall, the research contributes significantly to understanding migraine and informs clinical practice and policy development.

Limitations

The retrospective study design of this study may introduce recall bias and limit the accuracy of the data being collected. Additionally, the study was conducted in a single center which may limit the generalizability of the findings to other populations. The brief duration of the study may compromise the generalizability of its findings compared to studies conducted over an extended period of time. Furthermore, the study relied on self-reported data which may introduce reporting bias in data collection.

## Conclusions

This cross-sectional study provides valuable insights into the clinical features, treatment trends, and overall burden of episodic and chronic migraine in Tamil Nadu, India. It underscores the substantial impact of migraine on patient’s daily lives, with differences noted between those with episodic and chronic forms of the condition. The study highlights the prevalence of migraine triggers, comorbidities, and treatment patterns, revealing significant associations and disparities between the two migraine subtypes. Additionally, it emphasizes the importance of assessing disability and productivity using tools like the MIDAS questionnaire, with CM patients exhibiting higher disability scores compared to EM sufferers. These findings underscore the need for tailored management strategies, including preventive therapies and lifestyle modifications, to effectively address the diverse needs of migraine patients and alleviate their burden of illness. Further research is warranted to explore additional factors influencing migraine presentation and outcomes in this population.

## Figures and Tables

**Figure 1:**
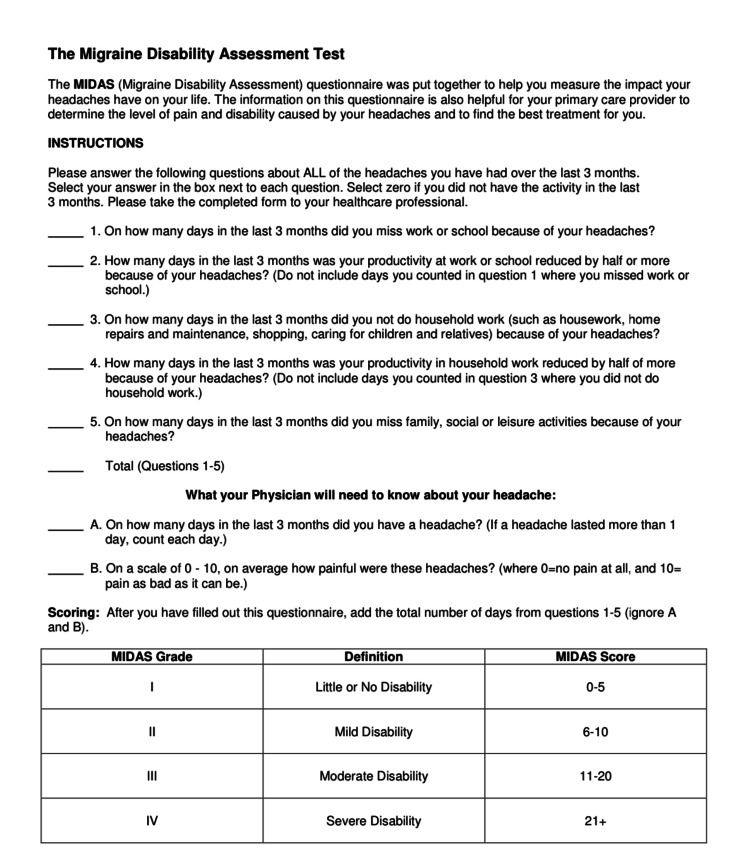
Migraine Disability Assessment Score (MIDAS) questionnaire Source: National Headache Foundation [5]

## Appendices

For information regarding the International Classification of Headache Disorder (ICHD-3) diagnostic criteria for migraine, kindly refer to this link: https://ichd-3.org/1-migraine/
